# Contributions of common and rare genetic variation to different measures of mood and anxiety disorder in the UK Biobank

**DOI:** 10.1192/bjo.2025.43

**Published:** 2025-05-09

**Authors:** Ioanna K. Katzourou, LINC Consortium, Inês Barroso, Lauren Benger, Andrés Ingason, Daniel Stow, Ruby Tsang, Megan Wood, George Kirov, James Walters, Michael J. Owen, Peter Holmans, Marianne B. M. van den Bree

**Affiliations:** Centre for Neuropsychiatric Genetics and Genomics, Cardiff University, Cardiff, UK; Medical School, University of Exeter, Exeter, UK; Institute of Biological Psychiatry, Roskilde, Denmark; Wolfson Institute for Population Health, Queen Mary University of London, London, UK; Bristol Medical School, University of Bristol, Bristol, UK; School of Psychology, University of Leeds, Leeds, UK; Neuroscience and Mental Health Innovation Institute Division of Psychological Medicine and Clinical Neurosciences, Cardiff University, Cardiff, UK

**Keywords:** Internalising disorders, depression, anxiety, UK Biobank, genetics

## Abstract

**Background:**

Mood and anxiety disorders co-occur and share symptoms, treatments and genetic risk, but it is unclear whether combining them into a single phenotype would better capture genetic variation. The contribution of common genetic variation to these disorders has been investigated using a range of measures; however, the differences in their ability to capture variation remain unclear, while the impact of rare variation is mostly unexplored.

**Aims:**

We aimed to explore the contributions of common genetic variation and copy number variations associated with risk of psychiatric morbidity (P-CNVs) to different measures of internalising disorders.

**Method:**

We investigated eight definitions of mood and anxiety disorder, and a combined internalising disorder, derived from self-report questionnaires, diagnostic assessments and electronic healthcare records (EHRs). Association of these definitions with polygenic risk scores (PRSs) of major depressive disorder and anxiety disorder, as well as presence of a P-CNV, was assessed.

**Results:**

The effect sizes of both PRSs and P-CNVs were similar for mood and anxiety disorder. Compared to mood and anxiety disorder, internalising disorder resulted in higher prediction accuracy for PRSs, and increased significance of associations with P-CNVs for most definitions. Comparison across the eight definitions showed that PRSs had higher prediction accuracy and effect sizes for stricter definitions, whereas P-CNVs were more strongly associated with EHR- and self-report-based definitions.

**Conclusions:**

Future studies may benefit from using a combined internalising disorder phenotype, and may need to consider that different phenotype definitions may be more informative depending on whether common or rare variation is studied.

Mood and anxiety disorders are highly prevalent, affecting over 300 million people worldwide,^1^ have a detrimental impact on the quality of life of affected individuals and those close to them^2^ and result in increased healthcare costs.^3,4^ Evidence from psychiatric genetics research indicates that currently used diagnostic boundaries do not accurately reflect the underlying shared genetic architecture of psychopathology.^5^ Research findings demonstrate that psychiatric conditions are highly polygenic, and that there is overlap in genetic risk between diagnostic groups.^6^

Mood and anxiety disorders tend to co-occur,^7^ respond to similar pharmacological and psychological treatments^8,9^ and have been indicated to involve similar neurobiological mechanisms.^10^ Despite extensive research, their aetiology is still incompletely understood; however, it is evident that genetic predisposition plays a role, with common^11–14^ as well as rare variants^15,16^ having been associated with both disorders. There is abundant evidence that the two disorders share genetic liability.^17–21^ There may therefore be benefits to conducting analyses where the two disorders are combined into a single internalising disorder phenotype, which may be better able to capture genetic variation than each disorder individually; however, to date there has been no published research examining this.

The contribution of common genetic variation to risk of psychiatric disorders is conferred by multiple variants, each of modest effect size, resulting in limited predictive power.^22^ Polygenic risk scores (PRSs) integrate the effect sizes of multiple variants throughout the genome and create quantifiable scores that are better suited for risk prediction than individual variants.^23^ In addition to common variants, a range of rare genetic variants, such as copy number variants (CNVs), have been reported to have a large effect on an individual’s risk of psychiatric outcomes.^24^ CNVs are rare sub-microscopic genomic re-arrangements, including deletions or duplications, and a range of these have been found to greatly increase risk of neurodevelopmental^25^ and psychiatric disorders (P-CNVs from here onwards). For example, these CNVs have been associated with elevated rates of anxiety disorders in youth^24^ and anxiety and mood disorders in adulthood.^15,16^ While both P-CNVs and common variations are implicated in the development of anxiety and mood disorders, the majority of the literature focuses exclusively on either common or rare variants, with few studies exploring both.^25^

Large case–control studies are required to detect the contributions of genetic variants to psychiatric disorders. The recruitment of patients in such studies is resource-intensive and can introduce biases inherent to the selection of participants.^26^ An alternative approach is utilising the breadth of phenotypic information that large-scale population-based biobanks can provide, where extensive sample sizes can contribute to the discovery of new genotype–phenotype associations. This is particularly the case for common disorders, as indicated by recent genome-wide association studies (GWASs) of major depressive disorder (MDD) in cohorts of very large size.^11,12^ However, the findings of this type of study crucially depend on the correct assignment of participants to case or control status, which will differ depending on the measures that have been selected out of those available in the biobank. It is likely that different phenotypic definitions differ in the ability to classify individuals into cases and controls, and therefore their ability to capture genetic variation. Definitions that are more sensitive to capturing disease-specific genetic variation will be more informative in identifying the biological pathways involved in these conditions and ultimately in guiding future intervention strategies. In the case of MDD, it has been reported that genetic analyses conducted on measures involving minimal phenotyping (e.g. self-reported seeking of medical attention or diagnosis) result not only in a reduced single-nucleotide polymorphism (SNP) heritability, but also in genetic associations that are less specific to MDD, showing greater overlap with other neuropsychiatric traits.^27^ In contrast, more strict, narrowly defined phenotypic measures were found to yield greater SNP heritability and to better capture MDD-specific genetic variation.^27^ This work did, however, not include some other commonly used measures of mood disorder, such as primary care records or medication use, while it also remains unclear if similar findings apply to anxiety disorders. Furthermore, it is unknown if these results extend to risk attributable to rare genetic variation.

The great majority of the psychiatric genetics literature to date has focused on individuals of European ancestry, with few studies examining individuals of different genetic ancestries.^28^ This has meant that individuals of non-European ancestries have been removed from data-sets before genetic analysis is undertaken. Novel analytical strategies are now providing opportunities to conduct genomic studies in ancestrally diverse and admixed populations, allowing for more inclusive and representative studies,^29,30^ increasing the generalisability of findings.

## Aims

The purpose of this study was to investigate the contributions of common genetic variation and P-CNVs to a range of self-reported and electronic healthcare record (EHR)-derived definitions of anxiety and mood disorders and to evaluate whether combining these disorders into an internalising disorder phenotype improves the ability to capture genetic influences. We focused on the UK Biobank (UKBB) cohort because it is a large population-based resource, combining information on anxiety and mood disorders from a range of different sources with genetic data.

Specifically, we aimed to achieve the following.

- Evaluate whether the predictive accuracy of PRSs for anxiety and MDD improves when information on anxiety and mood is combined into an internalising disorder phenotype, in comparison to analyses based on individual phenotypes of anxiety and mood disorder.
- Investigate the differences in association with these PRSs between eight different definitions for each of mood, anxiety and internalising disorder, based on self-report questionnaires, diagnostic interviews, medication use and primary care and hospital admission EHR data.
- Investigate if the presence of a P-CNV is more strongly associated with (i) the combined internalising disorder phenotype than with individual phenotypes of anxiety or mood disorder and (ii) any of the eight definitions for each of mood, anxiety and internalising disorder mentioned above in (b).

These analyses included participants in the UKBB of all ancestries and PRSs were adjusted for ancestral differences.

## Method

### Participants

The UKBB is a prospective study of over 500 000 individuals living in the UK.^31^ Participants aged between 40 and 69 years old were recruited between 2006 and 2010. They attended a baseline assessment as well as multiple repeat assessments. The UKBB received ethical approval from the North West - Haydock Research Ethics Committee (reference 16/NW/0274). Participants provided electronic signed consent at recruitment. This study was conducted under application number 79704.

### Phenotyping

Four main sources of information relevant to anxiety and mood disorder were identified in the UKBB. These were as follows:

- a touchscreen questionnaire completed by participants during initial recruitment to the study at recruitment centres;
- a nurse-led interview completed at recruitment to which participants were invited if they stated in the touchscreen questionnaire that they had been diagnosed with certain long-term conditions or were currently taking medication;
- linked EHRs, including hospital admission records (available for the whole cohort) and primary care records (available for ∼40% of the cohort);
- the mental health questionnaire (MHQ), which was an online follow-up sent to all participants with a valid email address.^7^

**Fig. 1:**
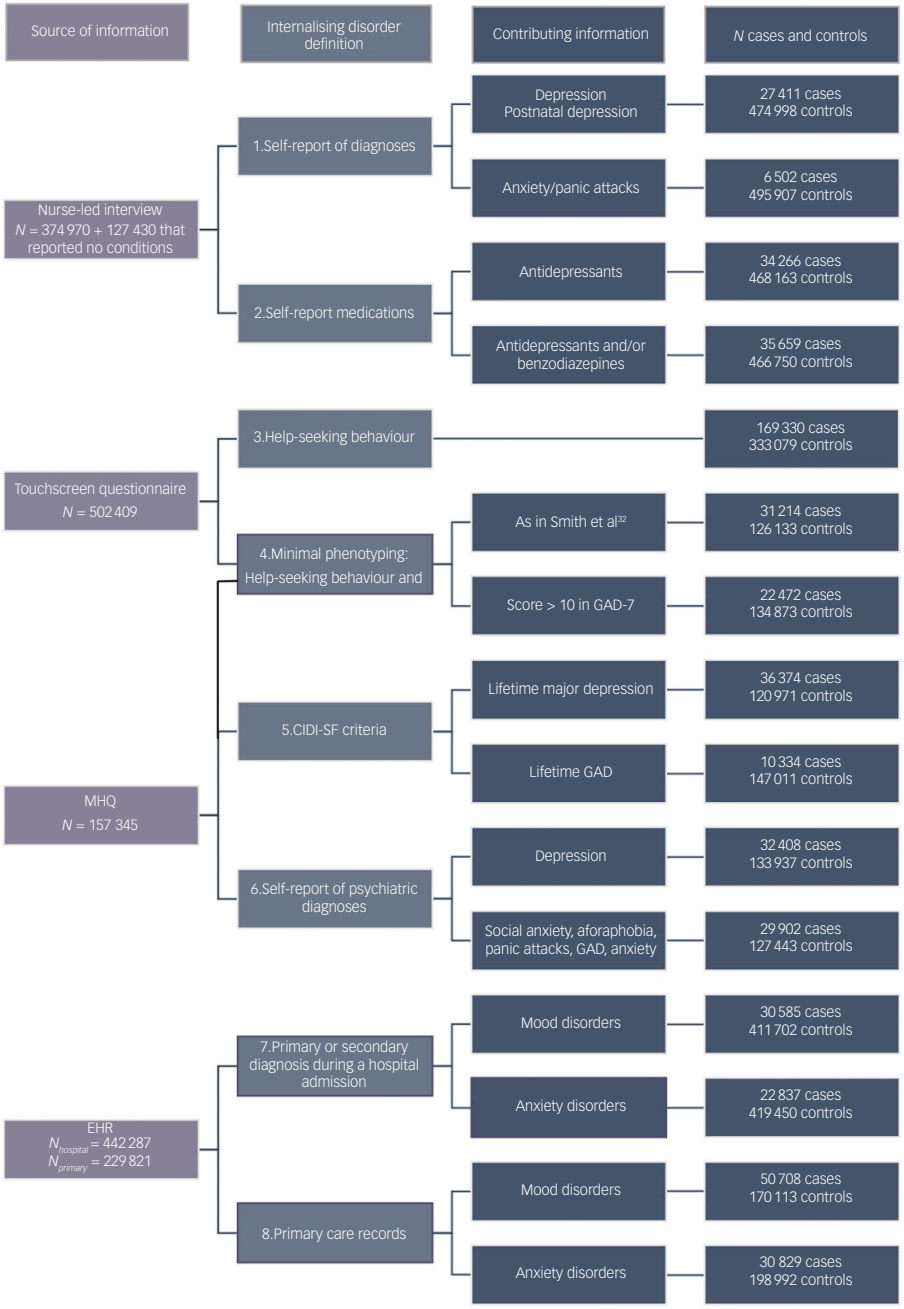
Data sources for internalising disorder definitions in the UK Biobank. Self-report (coded 1) was defined as having reported during the nurse-led interview a diagnosis of depression or postnatal depression for mood disorder and anxiety/panic attacks for anxiety disorder. Medication self-report (coded 2) was defined as having reported during the nurse-led interview currently being on a prescription of any antidepressant for mood disorder and any antidepressant and/or benzodiazepine apart from temazepam for anxiety disorder. Help-seeking behaviour (coded 3) was defined as having answered yes to either ‘have you ever seen a GP [general practitioner] for depression, tension or nerves?’ or ‘have you ever seen a psychiatrist for depression, tension or nerves?’, and thus help-seeking behaviour is identical for mood and anxiety disorders. Minimal phenotyping (coded 4 in Fig. 1) was defined according to Smith et al^32^ for mood disorder and as having endorsed the help-seeking phenotype and in addition having a score of 10 or above on the generalised anxiety disorder 7 (GAD-7)^33^ for anxiety disorder. The Composite International Diagnostic Interview Short-Form (CIDI-SF) (coded 5) was defined using items of the mental health questionnaire (MHQ) that correspond to the CIDI-SF^34^ diagnostic criteria for lifetime major depression for mood disorder and lifetime generalised anxiety disorder (GAD) for anxiety disorder. The MHQ self-report (coded 6) was defined as having reported in the MHQ having had a diagnosis of depression for mood disorder or social anxiety or social phobia, agoraphobia, panic attacks, anxiety, nerves and GAD for anxiety disorder. The presence of mood and anxiety disorder in hospital admission records (coded 7) and primary care records (coded 8) was established using lists of clinical codes curated by the MULTIPLY^35^ project and amended to exclude specific phobias and other non-specific codes (Supplementary Material). EHR, electronic healthcare record.

The numbers of individuals with available data for these four sources are summarised in Fig. 1. Using these sources of information, eight ways of defining internalising disorder were derived, summarised in Fig. 1. For each definition, individuals that were established to have either a mood or anxiety disorder or both were classified as having an internalising disorder.

Individuals who had a diagnosis of schizophrenia or bipolar disorder either recorded in EHRs or self-reported at the nurse-led interview or the MHQ (*n* = 4214) were excluded from the analyses, as these disorders often share symptomatology with and could be misdiagnosed as internalising disorders. Thus, a sample of maximum *n* = 496 412 was available for analysis.

### Genetic analyses

#### Genetic quality control

The UKBB had imputed genotype data to the Haplotype Reference Consortium and the UK10K Consortium using IMPUTE4 software (https://jmarchini.org/software/).^31^ Preliminary quality control of the genotype data had also been performed by the UKBB.^31^ We performed additional quality control using PLINK version 2.0 for Linux^36^ (https://www.cog-genomics.org/plink/2.0/) to filter for variants with a low INFO score (<0.9), high missingness (>0.05), low minor allele frequency (<0.01) or variants departing from Hardy–Weinberg equilibrium (*p* < 10^−6^) and individuals with high missingness (>0.05) or sex discordance. Sex chromosomes were excluded. Kinship estimates were computed to identify individuals related to the second degree (Kinship-based INference for GWAS (KING)^37^ *r* ^2^ > 0.0884), and one individual from each related pair was removed at random. After quality control, 449 646 individuals and 6 899 626 variants were retained.

#### Polygenic risk score generation

Anxiety disorder PRSs were calculated using summary statistics from the iPSYCH anxiety disorder GWAS^38^ (4584 cases, 19 225 controls). MDD PRSs were calculated using summary statistics from the latest Psychiatric Genomics Consortium (PGC) MDD GWAS,^12^ excluding the UKBB cohort (45 621 cases, 97 674 controls). PRS-CS^39^ was used for PRS calculation. PRS-CS is a Bayesian algorithm that can infer posterior effect sizes of SNPs via continuous shrinkage,^39^ therefore avoiding the need for linkage disequilibrium pruning and *p*-value thresholding. The inferred posterior effect sizes were used for PRS generation on PLINK 2.0.^36^

#### Post hoc PRS adjustment for ancestry

To produce PRSs that are on the same scale across individuals from different ancestries, we adjusted them for ancestral differences in mean and variance using the 1000Genomes data-set as reference, as described by Khan et al^30^ The UKBB and 1000Genomes^40^ data-sets were merged, retaining only variants present in both (*N* SNPs 4 944 504). The two data-sets were then pruned using PLINK^36^ –indep-pairwise 500 50 0.05, resulting in 567 216 retained variants. FlashPCA for Linux^41^ (https://github.com/gabraham/flashpca) was used to generate principal components in the 1000Genomes data-sets and UKBB participants were projected onto these components. PRSs were calculated for the 1000Genomes data-set as described above.

First, using the 1000 Genomes data-set, the PRSs were regressed against the first five principal components to generate coefficients and residuals. The coefficients and residual variance from these models were then used to produce ancestry adjusted PRSs.^30^ The raw PRSs were standardised by subtracting the mean of the predicted PRS and dividing by the residual variance. The same procedure was subsequently performed in the UKBB using the principal component projections. The distribution of the adjusted PRSs between different ancestries was visually inspected in both the 1000 Genomes and UKBB data-sets. The post hoc adjustment was performed using R version 4.2 for Linux (https://www.r-project.org).^42^

#### PRS association analysis

The association of each of the eight definitions of mood, anxiety and internalising disorder with the standardised PRS of anxiety disorder and MDD was tested using logistic regression, adjusting for gender, age and the first ten genetic principal components to account for population structure. The predictive accuracy of the PRS was estimated using the receiver operating characteristic area under the curve (AUC), calculated by the pROC package in R.^43^ The proportion of phenotypic variance explained by the PRS was estimated by comparing Nagelkerke’s pseudo *R* ^2^ of the full model (PRS and covariates) with the null model (covariates only). The effect sizes of the PRSs from the logistic regression models were compared between the different definitions of internalising disorder. Because of the considerable sample overlap between the definitions, equations 6 and 7 from Lin and Sullivan^44^ were used to calculate the variance of the effect size difference, and thence a *z*-score for testing the significance of the difference. To assess the presence of gender-specific genetic effects, we also conducted the logistic regression analysis described above for each of the eight definitions of internalising disorder while adding an interaction term between gender and PRS. The analysis was performed using R.^42^

#### CNV calling

The process of calling the CNVs in the UKBB is described in detail elsewhere.^45^ Briefly, calling was performed using PennCNV-Affy 1.0.3 protocols for Linux (https://penncnv.openbioinformatics.org/en/latest/user-guide/affy/).^46^ Samples were excluded if they carried 30 or more CNVs, had a waviness factor greater than 0.03 or less than –0.03, a SNP call rate lower than 96%, or log *R* ratio s.d. higher than 0.35, while CNVs were excluded if they were covered by fewer than 20 probes, had a density coverage of less than 1 probe per 20 000 base pairs or a confidence score lower than 10, resulting in 454 254 individuals with available CNV call data.

#### CNV association analysis

A set of 54 CNVs that have previously been associated with an increased risk of a psychiatric disorder^45^ (P-CNVs) were studied. CNVs that were observed fewer than five times were excluded, resulting in 33 P-CNVs that were included in all subsequent analyses. First, the association of each of the eight definitions of mood, anxiety and internalising disorder with the presence of any of the P-CNVs was assessed using logistic regression, adjusting for age, gender and the first ten genetic principal components to account for population structure. Then, the association of the eight definitions of internalising disorders with each individual P-CNV was assessed in the same way. The effect sizes of the presence of a P-CNV from the logistic regression models were compared between the different definitions of internalising disorder as described above. We also conducted regression analysis for each of the eight definitions conditioning anxiety disorder on mood disorder. The purpose of this analysis was to test whether the associations of P-CNVs with anxiety disorder were independent of those with mood disorder. Non-independence of these associations provides further rationale for combining mood and anxiety disorder into an internalising disorder phenotype, since this will increase power without losing associations specific to the rarer disorder (anxiety). Then, to assess the presence of gender-specific genetic effects, we also conducted the logistic regression analysis described above for each of the eight definitions of internalising disorder while adding an interaction term between gender and the presence of a P-CNV. Finally, we assessed the association of each of the eight definitions of internalising disorder with the presence of each of the P-CNVs individually using logistic regression, adjusting for age, gender and the first ten genetic principal components to account for population structure. Statistical analyses were performed using R.^42^

#### Joint analysis of PRS and CNV

To examine if PRS and P-CNV act independently or whether there is evidence common and rare variations interact to increase the risk of internalising disorder, logistic regression analyses were performed as described previously, including the main effects of PRS, P-CNV and an interaction term PRS*P-CNV. The statistical analyses were performed using R.^42^

## Results

### Summary statistics

**Fig. 2:**
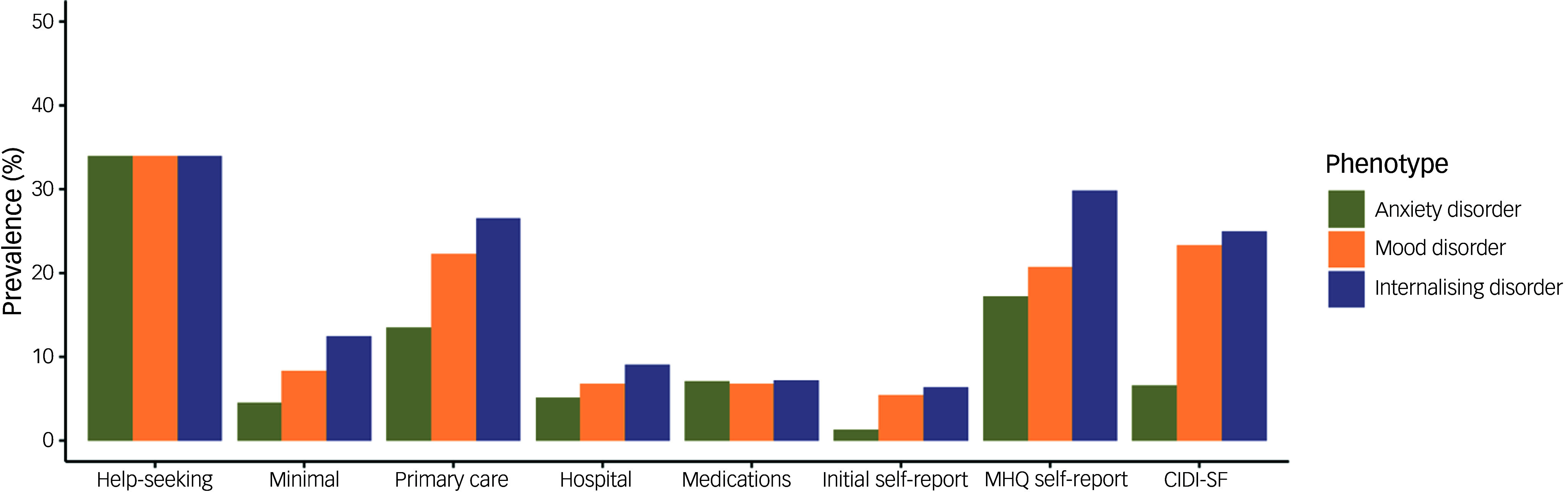
Prevalence of each definition of anxiety, mood and internalising disorder. For each definition, individuals with missing values were removed from the calculation. MHQ, mental health questionnaire; CIDI-SF, Composite International Diagnostic Interview Short-Form.

The prevalence of each definition of anxiety, mood and internalising disorder is shown in Fig. 2 and Supplementary Table 1 (available at https://doi.org/10.1192/bjo.2025.43). The frequency of each definition of internalising disorder for males and females is shown in Supplementary Fig. 1 and Supplementary Table 2. All definitions had a significantly higher prevalence in females compared to males. For the combined internalising disorder phenotype, the definition with the highest prevalence was help-seeking (33.7%, 169 330 cases). Because of the way this was queried in the touchscreen questionnaire, this definition was the same for mood, anxiety and internalising disorder (see Fig. 1). The lowest prevalence was found for initial self-report (7.74%, 31 869 cases). MHQ self-report and Composite International Diagnostic Interview Short-Form (CIDI-SF) had a high prevalence (29.57% and 24.71%, respectively), although the number of cases identified was not particularly large (46 514 and 38 887, respectively). For internalising disorder, the most common combinations of definitions are illustrated in Supplementary Fig. 2, while the number of definitions present for each individual is shown in Supplementary Fig. 3.

Tetrachoric correlations were calculated for each pair of phenotypes, as presented in Supplementary Fig. 4. All eight definitions of internalising disorder were significantly positively correlated.

### PRS analysis

The distributions of the PRS pre- and post-ancestry adjustment in the reference populations of the 1000Genomes data-set and in the UKBB populations (based on self-report of ethnicity) were visually examined. The distributions were notably different before adjustment, whereas post adjustment the shapes of the distributions were similar (Supplementary Figs. 5 and 6).

**Fig. 3:**
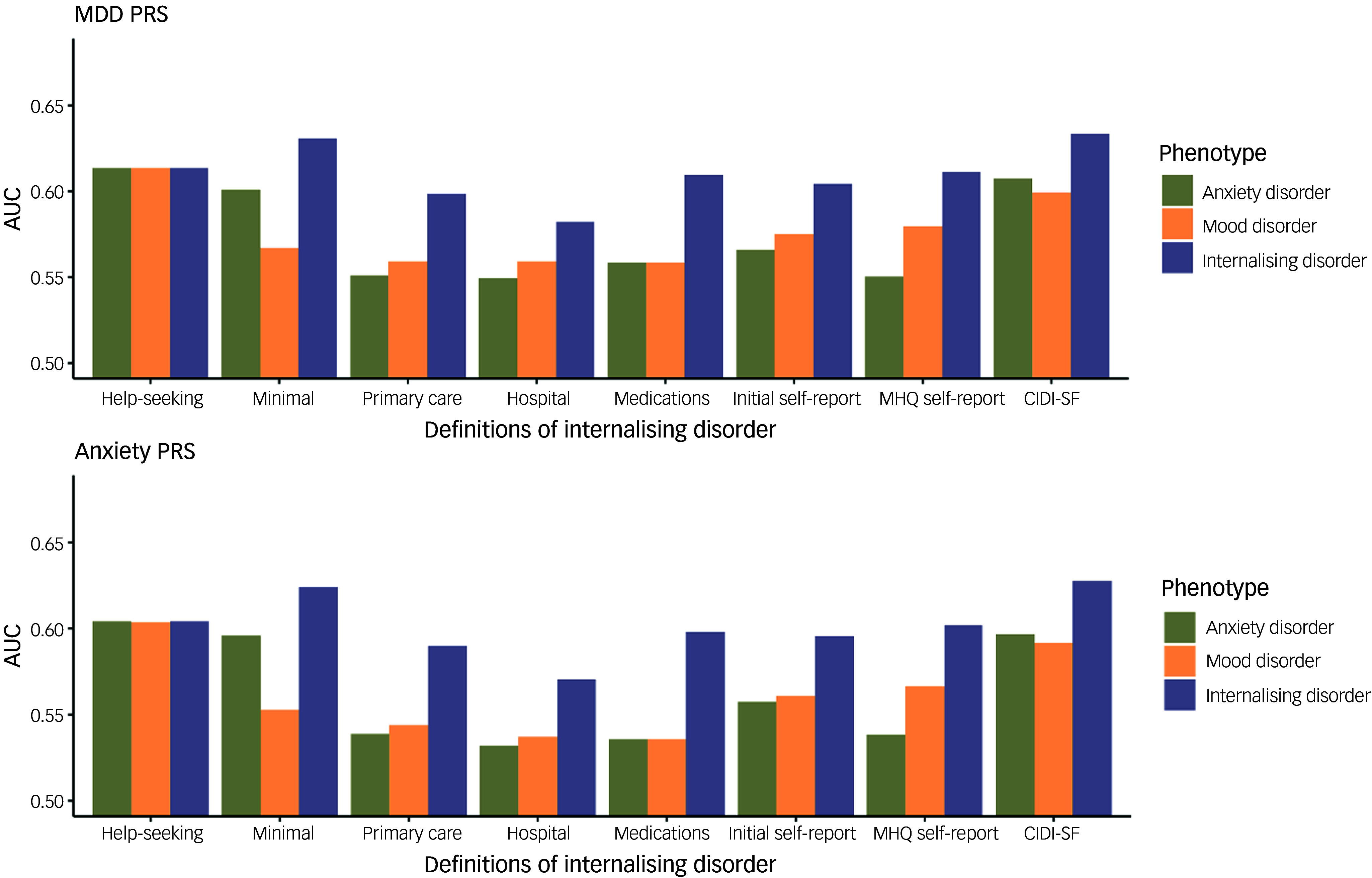
Prediction accuracy of major depressive disorder (MDD) (top) and anxiety disorder (bottom) polygenic risk scores (PRSs) for the eight definitions of mood disorders, anxiety disorders and internalising disorders. AUC, area under the curve; MHQ, mental health questionnaire; CIDI-SF, Composite International Diagnostic Interview Short-Form.

The association of the adjusted PRSs with the definitions of mood, anxiety and internalising disorder were assessed and the odds ratio with 95% confidence interval, *p*-value, Nagelkerke’s pseudo *R* ^2^ and AUC were calculated (Supplementary Table 3 for mood disorder, Supplementary Table 4 for anxiety disorder and Table 1 for internalising disorder). All phenotypes were significantly associated with both MDD and anxiety disorder PRS after Bonferroni correction for multiple testing, with the MDD PRS showing a more significant association and larger effect sizes for all phenotypes compared to the anxiety disorder PRS. The AUC of the models was used to quantify the prediction accuracy of the PRS for the different phenotypes, as illustrated in Fig. 3. Combining mood and anxiety disorder into internalising disorder resulted in increased AUC for both PRSs for all definitions, with the exception of help-seeking behaviour (which, as explained in the ‘Summary statistics’ section, is the only definition that does not distinguish between mood and anxiety disorder, and is therefore identical for all three conditions, including internalising disorder). For most definitions, the odds ratios of the association of each of the two PRSs with mood and anxiety disorder were similar (Supplementary Tables 3 and 4), while combined internalising disorder phenotype yielded a higher AUC than either mood or anxiety disorder separately. This indicates that combining the disorders results in increased prediction accuracy in PRS analyses.

**Table 1:** Association metrics of the adjusted major depressive disorder (MDD) and anxiety polygenic risk score (PRS) with the eight internalising disorder phenotypes

Definition	MDD PRS				Anxiety PRS			
	Odds ratio (95% CI)	*p*-value	AUC	*R* ^2^ (%)	Odds ratio (95% CI)	*p*-value	AUC	*R* ^2^ (%)
Help-seeking	1.19 (1.18–1.20)	<10^−300^	0.6135	0.80	1.09 (1.08–1.09)	7.57 × 10^−139^	0.6039	0.19
Minimal	1.20 (1.19–1.22)	1.16 × 10^−251^	0.6309	0.61	1.08 (1.07–1.09)	1.82 × 10^−49^	0.6241	0.12
Primary care	1.18 (1.17–1.19)	9.93 × 10^−211^	0.5985	0.68	1.08 (1.07–1.09)	4.15 × 10^−52^	0.5901	0.16
Hospital	1.19 (1.18–1.21)	1.47 × 10^−198^	0.5822	0.51	1.09 (1.07–1.10)	4.08 × 10^−48^	0.5702	0.12
Medications	1.20 (1.19–1.22)	2.13 × 10^−198^	0.6098	0.50	1.10 (1.08–1.10)	3.21 × 10^−54^	0.5982	0.13
Initial self-report	1.20 (1.19–1.22)	2.36 × 10^−177^	0.6044	0.48	1.09 (1.07–1.10)	4.59 × 10^−41^	0.5957	0.10
MHQ self-report	1.20 (1.19–1.22)	3.61 × 10^−196^	0.6111	0.88	1.08 (1.07–1.09)	5.68 × 10^−37^	0.6018	0.16
CIDI-SF	1.18 (1.17–1.20)	5.30 × 10^−150^	0.6336	0.69	1.07 (1.05–1.08)	3.68 × 10^−24^	0.6274	0.10

AUC, area under the curve; *R*^2^, Nagelkerke’s pseudo *R*^2^; MHQ, mental health questionnaire; CIDI-SF, Composite International Diagnostic Interview Short-Form.

The effect sizes (odds ratios) for each of the PRSs did not differ significantly between the definitions. The highest predictive accuracy (AUC) for both PRSs was observed for the CIDI-SF definition. The AUC was highest for the MHQ-derived phenotypes (CIDI-SF and minimal phenotyping), while the AUC was lowest for EHR-derived phenotypes, with the AUC for help-seeking behaviour and the self-reported phenotypes being in between. The Nagelkerke’s pseudo *R* ^2^ was low, ranging between 0.50% and 0.88% for the MDD PRS and 0.10–0.19% for the anxiety PRS. Age and female gender were significantly positively associated with all the definitions. We found no significant interaction between gender and either of the PRSs.

Restricting the analyses to individuals of European ancestry (self-reported ethnicity White British, White Irish or any other White background, *n* = 418 120) gave similar association results to those of the full cohort (Supplementary Table 5). Restricting to individuals of non-European ancestry (all other self-reported ethnicities, *n* = 25 782, Supplementary Table 6) led to lower odds ratios than in the European ancestry sample, although the differences were not significant. Associations with PRS also yielded less significant *p*-values than in the analyses of European ancestry (Supplementary Table 7), with *p*-values not meeting the significance threshold for anxiety PRS (Bonferroni-corrected *p*-value threshold 3.125 × 10^−3^, *n* tests 16) for many phenotypic definitions. Comparison of association of ethnicity-adjusted versus unadjusted PRS in individuals of non-European ancestry indicated that adjusted PRSs resulted in higher odds ratios; however, the difference was not significant (Supplementary Table 7).

### CNV analysis

The total number of individuals with a P-CNV was 7454. The numbers of individuals with a P-CNV endorsing each of the definitions of mood, anxiety and internalising disorder are shown in Table 2. The associations between presence of any of the P-CNVs and the definitions of mood, anxiety and internalising disorder are shown in Table 2. For most of the definitions, the odds ratios were similar between mood, anxiety and internalising disorder, and using the combined internalising disorder definitions resulted in similar odds ratios and increased significance. None of the associations between P-CNV and anxiety disorder were significant after conditioning on mood disorder. This highlights the interdependency of the mood and anxiety disorder phenotypes and provides further rationale for combining them into an internalising disorder phenotype to increase power without losing associations specific to the rarer disorder (anxiety). Of note, the only negative association in Table 2, between the presence of a P-CNV and the minimal definition for anxiety disorder was no longer significant after controlling for mood disorder.

**Table 2:** Results of the logistic regression of copy number variations associated with risk of psychiatric morbidity (P-CNV) carrier status with the eight definitions of mood, anxiety and internalising disorder and number of individuals with a P-CNV and each definition of mood disorder, anxiety disorder and internalising disorder

Definition	Anxiety disorder		Cases with P-CNV	Mood disorder		Cases with P-CNV	Internalising disorder		Cases with P-CNV
	Odds ratio (CI)	*p*-value		Odds ratio (CI)	*p*-value		Odds ratio (CI)	*p*-value	
Help-seeking	1.08 (1.03–1.14)	1.54 × 10^−3^	2665	1.08 (1.03–1.14)	1.54 × 10^−3^	2665	1.08 (1.03–1.14)	1.54 × 10^−3^	2665
Minimal	0.81 (0.72–0.93)	7.98 × 10^−4^	473	1.03 (0.93–1.13)	0.574	473	0.95 (0.88–1.03)	0.229	691
Primary care	1.20 (1.09–1.31)	1.38 × 10^−4^	901	1.22 (1.13–1.31)	6.84 × 10^−7^	901	1.22 (1.13–1.31)	1.21 × 10^−7^	1066
Hospital	1.32 (1.19–1.46)	5.25 × 10^−8^	601	1.34 (1.23–1.46)	2.18 × 10^−11^	601	1.31 (1.22–1.43)	3.59 × 10^−12^	775
Medications	1.26 (1.16–1.36)	6.05 × 10^−8^	629	1.26 (1.16–1.37)	7.94 × 10^−8^	629	1.24 (1.15–1.35)	1.68 × 10^−8^	658
Initial self-report	1.16 (0.97–1.39)	0.120	526	1.28 (1.16–1.40)	1.54 × 10^−7^	526	1.26 (1.16–1.37)	1.22 × 10^−7^	605
MHQ self-report	1.24 (1.11–1.39)	1.52 × 10^−4^	455	1.14 (1.03–1.28)	0.013	455	1.18 (1.07–1.30)	8.07 × 10^−4^	652
CIDI-SF	1.19 (1.00–1.40)	0.042	484	1.06 (0.96–1.18)	0.244	2665	1.06 (0.95–1.17)	0.275	515

MHQ, mental health questionnaire; CIDI-SF, Composite International Diagnostic Interview Short-Form.

When correcting for multiple testing (Bonferroni-corrected *p*-value threshold 2.083 × 10^−3^, *n* tests 24), the presence of a P-CNV was significantly associated with six of the definitions of internalising disorders, but not with the CIDI-SF or minimal phenotyping. The highest effect sizes were observed for EHR-derived and self-reported definitions (initial self-report and medication self-report). The odds ratio for help-seeking behaviour was significantly lower than those for all other definitions that had a significant association with the presence of a P-CNV, while the odds ratio for hospital admission records was significantly higher than those for primary care records and MHQ self-report, but not initial self-report and medication self-report, and there was no significant difference between the odds ratios for MHQ self -report, primary care records, initial self-report and medication self-report. Minimal and CIDI-SF were not significantly associated with the presence of a P-CNV; therefore, the odds ratios for these definitions were not included in the comparisons. The *p*-values of the pairwise odds ratio comparisons are given in Supplementary Table 8. We found no significant interaction between gender and the presence of a P-CNV for any of the outcomes.

We subsequently examined the association of the 38 P-CNVs with the definitions of internalising disorders individually. The numbers of individuals with each of the 38 P-CNVs are shown in Supplementary Table 9. The results are illustrated in Supplementary Fig. 7. The pattern of association of individual P-CNVs with the eight definitions of internalising disorders was complex, with no individual P-CNV showing association with all definitions. For some of the individual P-CNVs the pattern of association was similar to that of the aggregated P-CNVs, for example, 15q11.2 duplication and 16p13.11 deletion were significantly positively associated with EHR-derived and self-reported definitions and not with MHQ- and questionnaire-based definitions. On the other hand, 17p13.3 duplication and 22q11.2 deletion were positively associated with CIDI-SF and MHQ self-report, while 22q11.2 distal deletion and 15q24 duplication were negatively associated with CIDI-SF and MHQ self-report, findings that were in contrast to the aggregated P-CNV results. No P-CNVs were associated with help-seeking behaviour.

### PRS and CNV interaction analysis

No evidence of significant interaction of either PRS with presence of a P-CNV was found for any of the definitions of internalising disorders (Bonferroni-corrected *p*-value threshold 3.125 × 10^−3^, *n* tests 16). The results are shown in Supplementary Table 10.

## Discussion

Our study aimed to explore the genetic architecture of internalising disorders and to assess the genetic burden associated with different definitions of these disorders derived from a number of different types of assessments. We hypothesised that mood and anxiety disorder can be grouped into a combined internalising disorder phenotype, based on previous work that has illustrated that the two disorders correlate phenotypically and genetically.^19–21^ We constructed eight different phenotypic definitions of mood, anxiety and internalising disorder, aiming to determine if there are ways of defining the disorder that better capture genetic liability. We aimed to examine the effect of both common and rare genetic variation across the genome and included UKBB participants of all ethnic backgrounds. We found that combining mood and anxiety disorder into an internalising disorder resulted in a higher predictive accuracy in PRS analyses, regardless of the way in which the phenotype data were obtained. For P-CNVs, we found that combining the disorders resulted in similar or higher effect sizes and stronger associations for some of the definitions. Moreover, we found that stricter definitions of internalising disorders resulted in better prediction accuracy in PRS analyses, while EHR-derived and self-reported definitions had the highest effect sizes in analysis of P-CNVs.

We combined information on mood and anxiety disorder into a single internalising disorder and compared the association of PRS derived from GWAS of MDD^12^ and anxiety disorder^38^ on this phenotype with those for mood and anxiety disorder measured individually. Anxiety and mood disorder had similar associations with each of the two PRSs. The combined internalising disorder phenotype resulted in similar or higher effect sizes, more significant associations and higher predictive accuracy than the mood and anxiety disorder definitions individually. This was the case across all eight disorder definitions. While the increased significance could result from the higher number of affected individuals for internalising disorder, the higher AUCs would indicate that the strengthening of the results also stems from the genetic overlap between anxiety and mood disorder. The anxiety disorder PRS had a lower odds ratio and AUC than the MDD PRS across all eight definitions, even when predicting anxiety disorder. However, the GWAS used to derive the anxiety PRS had a smaller sample size,^12^ and thus the anxiety PRS is likely to be less powerful than the MDD PRS. The AUCs we found ranged from 0.75 to 0.63, similar to those reported in the literature for depression PRS (0.57),^12^ bipolar disorder PRS (0.65),^47^ schizophrenia PRS (0.72)^48^ and Alzheimer’s disease PRS (0.69).^49^

When comparing the association of the PRSs with the eight definitions of internalising disorder, both PRSs had a higher prediction accuracy for MHQ-derived definitions of internalising disorders that include standardised questionnaires, such as the CIDI-SF and minimal phenotyping. These definitions are totally or partially based on parts of the MHQ.^7^ EHR-derived definitions, such as primary care records and hospital admissions, showed the lowest prediction accuracy. This is in agreement with earlier investigations of the genetic liability of different definitions of depression in the UKBB that have found that depression diagnosed using the CIDI-SF has the highest SNP heritability and help-seeking behaviour the lowest.^27,50^ Cai et al^27^ found that the genetic liability of minimally defined depression, a phenotype similar to the help-seeking behaviour used in this study, is less specific to depression and includes more liability shared with other psychiatric traits. In the same study, the PRS derived from help-seeking behaviour had the highest prediction accuracy for depression in a separate sample. However, when deriving a PRS from each definition using the same sample size, a CIDI-based definition of depression resulted in the highest AUC.^50^ When we derived the PRS of MDD and tested its association with the different definitions, we also found the highest prediction accuracy was achieved when using CIDI-SF definitions. We have, therefore, shown that conclusions regarding the genetic liability of different definitions of depression also extend to definitions of anxiety and combined internalising disorder. In addition, our study included a wider range of phenotypic definitions that are often used in public health studies, including primary care records and medication use. Interestingly, we found that primary care records, hospital admission records and self-reported medication use had the lowest AUC for both MDD and anxiety PRSs, indicating that they capture common genetic variation less well than the other definitions we studied.

We compared UKBB participants with at least one of 38 CNVs previously associated with high risk of a psychiatric condition^45^ with those without these P-CNVs. We assessed the association of the presence of these P-CNVs with the different definitions of mood, anxiety and internalising disorder. The odds ratios for anxiety and mood disorder were similar, and combining the disorders resulted in more significant associations for some of the definitions as it increased the statistical power because of an increase in the number of affected individuals. Three of the definitions (initial self-report, CIDI-SF and minimal) were endorsed by fewer individuals with a P-CNV for anxiety disorder than for mood disorder; therefore, for these the association with internalising disorder was likely mostly driven by mood disorder.

We found the presence of a P-CNV had the highest effect size for EHR-derived definitions (primary care and hospital admission records) and definitions based on the self-report at recruitment (initial self-report and medication self-report). The CIDI-SF and minimal phenotyping were not associated with the presence of a P-CNV. This is in direct contrast to the results of the PRS analyses. A possible explanation could be that internalising disorders caused by common genetic variation are less severe, and therefore less likely to result in the use of healthcare services compared to internalising disorders that are associated with a rare genetic variant. On the other hand, individuals with a P-CNV have been found to have a higher risk of developing physical and mental health multimorbidity,^51,52^ and it is therefore likely that they have more contact with health services than individuals without these CNVs. This could mean that any evidence of internalising disorder is also more likely to be queried and diagnosed by a physician and treated, and therefore recorded in their EHR or self-reported as a diagnosis or a medication. Finally, the presence of an interactive effect between PRS and the presence of a P-CNV was explored. There was no significant interaction between the presence of a P-CNV and either of the PRSs for any of the definitions of internalising disorder, which is in agreement with recent findings of the effect of common and rare variation on psychopathology in the UKBB.^25^ This suggests that the risk conferred by common and rare genetic variants is independent, at least for the definitions of internalising disorder examined. Interestingly, we found no significant interaction between gender and any of the genetic risk factors we examined, which indicates that these do not act in a gender-specific way.

While the majority of studies into the genetics of mental health conditions have been restricted to a single population, usually comprising of individuals of European ancestry,^28^ we aimed to include the whole UKBB cohort in our study and not restrict our analysis to a single ethnic background, as including ethnically diverse populations in genetic studies can uncover differing biological risk factors and aid in combating health inequalities. As PRSs derived from European cohorts have been found to perform sub-optimally in populations of non-European ancestry,^53^ we attempted to adjust the PRS for ancestry effects using an ancestrally diverse data-set as our reference. Before adjustment, there were considerable differences in the mean and variance of both MDD and anxiety disorder PRSs; post-adjustment, however, the PRS distributions were notably more aligned, particularly so for the MDD PRS. While the adjustment did not result in perfect alignment of the distributions, this method allowed for including UKBB participants of all ancestries in the analysis.

There are limitations to this study. First, the MDD GWAS that was used for PRS generation in this analysis was based on a larger sample size^12^ and thus better captured the genetic risk than the anxiety disorder GWAS.^38^ However, the two disorders are genetically and phenotypically correlated,^6,19^ and our results indicate that the MDD PRS also captures genetic risk for anxiety disorders. Moreover, while the UKBB is one of the largest population cohorts with genetic data available worldwide and contains rich phenotypic information, it has been found to be affected by selection bias, with participants having better health and higher socioeconomic status than the general population in the UK.^54^ In addition, the UKBB was designed as a prospective population cohort study of middle and older age,^31^ recruiting participants between 40 and 69 years of age, which makes it susceptible to survival bias. Internalising disorders are associated with premature mortality,^55^ and therefore it is likely that individuals with the most severe manifestations of these disorders would not be included in such a cohort. Finally, UKBB participants who have completed the MHQ have been found to be of higher socioeconomic status and better overall health than the average UKBB participant, with a further bias towards individuals of European descent.^7^ This is particularly important for the CNV analyses, as the number of individuals with a P-CNV that completed the MHQ was low (1950, 26.16% of individuals with a P-CNV, compared to 31.61% completion for individuals without a P-CNV), and therefore the sample size might not have been sufficient to uncover significant associations with these phenotypes.

In conclusion, this study aimed to explore the genetic architecture of internalising disorder definitions. Our results indicate that combining mood and anxiety disorders into an internalising disorder phenotype can be of benefit in genetic analyses looking at both common and rare variants. The optimal definition of internalising disorders for use in genetic studies depends on the type of genetic researchers aim to uncover. While more clinically robust definitions of internalising disorders, such as the CIDI-SF diagnostic criteria, seem preferable when examining common variation, using EHR- or self-report-based definitions might be the optimal choice when rare variation is of interest.

## Supporting information

Katzourou et al. supplementary material Katzourou et al. supplementary material

## Data Availability

Relevant data is available from the UK Biobank subject to standard procedures (www.ukbiobank.ac.uk).

## Supplementary material

The supplementary material is available online at https://doi.org/10.1192/bjo.2025.43
