# Supplementary material for: Contributions of common and rare genetic variation to different measures of mood and anxiety disorder in the UK Biobank
Source: BJPsych Open. 2025 May 9;11(3):e97. doi: 10.1192/bjo.2025.43 (PMC12089803; doi:10.1192/bjo.2025.43)
Supplement: Katzourou et al. supplementary material [file S2056472425000432sup001.docx]

**SUPPLEMENTARY MATERIAL**

**SUPLLEMENTARY TABLES**

|  | Mood disorder | Anxiety  disorder | Internalising disorders |
| --- | --- | --- | --- |
| Help-seeking | 169,330 (33.70%) | 169,330 (33.70%) | 169,330 (33.70%) |
| Minimal | 31,214 (19.84%) | 22,472 (14.28%) | 48,819 (31.03%) |
| Primary care | 50,708 (22.06%) | 30,829 (13.41%) | 60,490 (26.32%) |
| Hospital | 30,585  (6.92%) | 22,837  (5.16%) | 40,753  (9.21%) |
| Medications | 34,266  (9.14%) | 35,659  (9.51%) | 36,244  (9.83%) |
| Initial self-report | 27,411  (7.31%) | 6,502  (1.73%) | 31,869  (7.74%) |
| MHQ self-report | 32,408 (20.60%) | 26,902 (17.10%) | 46,514 (29.57%) |
| CIDI-SF | 36,374 (23.12%) | 10,334  (6.57%) | 38,887 (24.71%) |

Supplementary Table 1. Number of individuals endorsing each definition of mood, anxiety and internalising disorder and prevalence of each definition. For each definition, individuals with missing values were removed from the prevalence calculation.

.

|  | Total |  | Cases |  | Prevalence |  |
| --- | --- | --- | --- | --- | --- | --- |
|  | Female | Male | Female | Male | Female | Male |
| Help-seeking | 273,325 | 269,710 | 109,863 | 58,249 | 40.7% | 25.8% |
| Minimal | 88,141 | 67,514 | 32,595 | 15,984 | 36.9% | 23.7% |
| Primary care | 123,980 | 102,727 | 39,073 | 21,157 | 31.5% | 20.6% |
| Hospital | 239,651 | 196,913 | 25,654 | 14,093 | 10.7% | 7.2% |
| Medications | 270,157 | 226,255 | 24,598 | 11,108 | 9.1% | 4.9% |
| Initial self-report | 270,157 | 226,255 | 20,931 | 10,564 | 7.8% | 4.7% |
| MHQ self-report | 88,141 | 67,514 | 31,290 | 15,121 | 35.5% | 22.4% |
| CIDI-SF | 88,141 | 67,514 | 26,714 | 12,086 | 30.3% | 17.9% |

Supplementary Table 2. Number of individuals endorsing each definition and prevalence of internalising disorders for each definition. For each definition, individuals with missing values were removed from the prevalence calculation.

|  | MDD PRS | |  | | Anxiety PRS | |  | |
| --- | --- | --- | --- | --- | --- | --- | --- | --- |
|  | **OR**  **(95% CI)** | **p-value** | | **AUC** | **OR**  **(95% CI)** | **p-value** | | **AUC** |
| Help-seeking | 1.19  (1.18-1.20) | <10^-300^ | | 0.6133 | 1.09  (1.08-1.09) | 7.57x10^-139^ | | 0.6036 |
| Minimal | 1.20  (1.18-1.21) | 2.71x10^-160^ | | 0.5670 | 1.08  (1.06-1.09) | 3.14x10^-31^ | | 0.5528 |
| Primary care | 1.19  (1.17-1.20) | 9.43x10^-199^ | | 0.5594 | 1.08  (1.07-1.09) | 7.84x10^-43^ | | 0.5440 |
| Hospital | 1.20  (1.19-1.22) | 2.15x10^-169^ | | 0.5591 | 1.09  (1.07-1.10) | 8.85x10^-39^ | | 0.5371 |
| Medications | 1.20  (1.19-1.22) | 1.51x10^-192^ | | 0.5582 | 1.10  (1.08-1.11) | 6.57x10^-54^ | | 0.5356 |
| Initial self-report | 1.21  (1.19-1.22) | 5.72x10^-179^ | | 0.5752 | 1.09  (1.07-1.10) | 1.08x10^-34^ | | 0.5610 |
| MHQ self-report | 1.21  (1.19-1.22) | 3.12x10^-160^ | | 0.5797 | 1.08  (1.06-1.09) | 1.26x10^-24^ | | 0.5665 |
| CIDI-SF | 1.18  (1.17-1.20) | 6.67x10^-136^ | | 0.5993 | 1.07  (1.06-1.08) | 3.23x10^-24^ | | 0.5915 |

Supplementary Table 3. Association metrics of the adjusted MDD and anxiety PRS with the eight definitions of mood disorder.

|  | MDD PRS | |  | | Anxiety PRS | |  | |
| --- | --- | --- | --- | --- | --- | --- | --- | --- |
|  | **OR**  **(95% CI)** | **p-value** | | **AUC** | **OR**  **(95% CI)** | **p-value** | | **AUC** |
| Help-seeking | 1.19  (1.18-1.20) | <10^-300^ | | 0.6135 | 1.09  (1.08-1.09) | 7.57x10^-139^ | | 0.6036 |
| Minimal | 1.15  (1.13-1.17) | 3.94x10^-77^ | | 0.6012 | 1.06  (1.04-1.07) | 6.14x10^-14^ | | 0.5960 |
| Primary care | 1.16  (1.15-1.18) | 1.05x10^-108^ | | 0.5512 | 1.09  (1.07-1.10) | 2.89x10^-37^ | | 0.5368 |
| Hospital | 1.17  (1.15-1.29) | 9.00x10^-94^ | | 0.5496 | 1.08  (1.06-1.09) | 2.86x10^-23^ | | 0.5320 |
| Medications | 1.20  (1.19-1.22) | 8.56x10^-199^ | | 0.5582 | 1.10  (1.08-1.11) | 1.42x10^-54^ | | 0.5356 |
| Initial self-report | 1.17  (1.14-1.21) | 7.96x10^-31^ | | 0.5659 | 1.10  (1.07-1.13) | 6.85x10^-13^ | | 0.5574 |
| MHQ self-report | 1.16  (1.14-1.18) | 2.05x10^-87^ | | 0.5504 | 1.08  (1.07-1.10) | 4.66x10^-28^ | | 0.5382 |
| CIDI-SF | 1.21  (1.18-1.24) | 1.76x10^-63^ | | 0.6075 | 1.07  (1.05-1.09) | 4.49x10^-10^ | | 0.5969 |

Supplementary Table 4. Association metrics of the adjusted MDD and anxiety PRS with the eight definitions of anxiety disorder.

|  | MDD PRS | | | Anxiety PRS | | |
| --- | --- | --- | --- | --- | --- | --- |
|  | **OR** | **p-value** | **AUC** | **OR** | **p-value** | **AUC** |
| Help-seeking | 1.19 | <10^-300^ | 0.6094 | 1.08 | 5.97x10^-137^ | 0.5990 |
| Minimal | 1.20 | 1.27x10^-246^ | 0.6279 | 1.08 | 9.52x10^-48^ | 0.6206 |
| Primary care | 1.18 | 3.37x10^-209^ | 0.5978 | 1.08 | 1.18x10^-54^ | 0.5892 |
| Hospital | 1.19 | 3.16x10^-190^ | 0.5806 | 1.08 | 1.34x10^-44^ | 0.5682 |
| Medications | 1.19 | 1.01x10^-192^ | 0.6059 | 1.09 | 2.55x10^-53^ | 0.5939 |
| Initial self-report | 1.19 | 4.29x10^-170^ | 0.5951 | 1.08 | 6.90x10^-40^ | 0.5901 |
| MHQ self-report | 1.20 | 2.31x10^-193^ | 0.6093 | 1.08 | 5.32x10^-37^ | 0.5998 |
| CIDI-SF | 1.18 | 2.11x10^-147^ | 0.6328 | 1.06 | 5.70x10^-24^ | 0.6265 |

Supplementary Table 5. Association metrics of the adjusted MDD and anxiety PRS with the eight definitions of internalising disorders in individuals of European ancestry.

|  | Total N | With NDD CNV |
| --- | --- | --- |
| Total | 25,935 | 296 |
| Help-seeking | 6,654 | 67 |
| Minimal | 2,030 | 13 |
| Primary care | 2,360 | 37 |
| Hospital | 1,837 | 26 |
| Medications | 1,284 | 14 |
| Initial self-report | 1,153 | 17 |
| MHQ self-report | 1,111 | 4 |
| CIDI-SF | 1,029 | 9 |

Supplementary Table 6. Number of individuals of non-European ancestry with each of the definitions of internalising disorder.

|  | Unadjusted  PRS | | | |  | Adjusted PRS | | |  |
| --- | --- | --- | --- | --- | --- | --- | --- | --- | --- |
|  | MDD PRS | | Anxiety PRS | | | MDD PRS | | Anxiety PRS | |
|  | **OR** | **p-value** | **OR** | **p-value** | | **OR** | **p-value** | **OR** | **p-value** |
| Help-seeking | 1.11 | 2.51x10^-12^ | 1.04 | 2.04x10^-3^ | | 1.14 | 1.48x10^-12^ | 1.07 | 2.61x10^-3^ |
| Minimal | 1.13 | 2.83x10^-7^ | 1.07 | 6.32x10^-3^ | | 1.16 | 1.62x10^-7^ | 1.11 | 4.68x10^-3^ |
| Primary care | 1.09 | 5.90x10^-4^ | 1.01 | 0.833 | | 1.11 | 1.82x10^-4^ | 1.01 | 0.811 |
| Hospital | 1.15 | 2.70x10^-8^ | 1.07 | 7.60x10^-3^ | | 1.19 | 1.20x10^-8^ | 1.12 | 2.11x10^-3^ |
| Medications | 1.16 | 7.03x10^-7^ | 1.05 | 0.077 | | 1.12 | 2.72x10^-7^ | 1.09 | 0.067 |
| Initial self-report | 1.19 | 3.71x10^-8^ | 1.06 | 0.052 | | 1.23 | 2.69x10^-8^ | 1.10 | 0.045 |
| MHQ self-report | 1.14 | 2.48x10^-4^ | 1.03 | 0.357 | | 1.17 | 1.71x10^-4^ | 1.05 | 0.421 |
| CIDI-SF | 1.14 | 4.37x10^-4^ | 1.04 | 0.279 | | 1.17 | 4.21x10^-4^ | 1.06 | 0.319 |

Supplementary Table 7. Association metrics of the ancestry adjusted and unadjusted MDD and anxiety PRS with the eight definitions of internalising disorders in individuals of non-European ancestry.

|  | Help-seeking |  |  |  |  |
| --- | --- | --- | --- | --- | --- |
| Primary care | 1.27x10^-11^ | **Primary care** |  |  |  |
| Hospital | 2.73x10^-26^ | 1.12x10^-3^ | **Hospital** |  |  |
| Medications | 1.96x10^-13^ | 0.398 | 0.143 | **Medications** |  |
| Initial self-report | 7.87x10^-15^ | 0.186 | 0.371 | 0.427 | **Initial self-report** |
| MHQ self-report | 9.80x10^-5^ | 0.198 | 7.15x10^-5^ | 0.325 | 0.526 |

Supplementary Table 8. P-values of pairwise comparisons of effect sizes of P-CNV between the definitions of internalising disorder. The two definitions of internalising disorder that were not significantly associated with the presence of a P-CNV (Minimal and CIDI-SF) were not included in this analysis

| CNV | Count |
| --- | --- |
| Any | 7,549 |
| TAR_del | 80 |
| TAR_dup | 463 |
| 1q21.1del | 119 |
| 1q21.1dup | 193 |
| NRXN1del | 176 |
| 2q11.2del | 34 |
| 2q13del | 57 |
| 2q13dup | 73 |
| 3q29del | 9 |
| WBS_dup | 16 |
| 8p23.1dup | 16 |
| 15q11.2del | 1,748 |
| 5q11.2dup | 2,284 |
| PWS_dup | 19 |
| 15q13.3del | 47 |
| 22q11.2distal_dup | 14 |
| 15q24dup | 9 |
| 16p13.11del | 140 |
| 16p13.11dup | 888 |
| 16p12.1del | 260 |
| 16p11.2distal_del | 62 |
| 16p11.2distal_dup | 143 |
| 16p11.2del | 123 |
| 6p11.2dup | 142 |
| 17p13.3_YWHAEdel | 27 |
| 17p13.3_YWHAEdup | 8 |
| Potocki_Lupski | 6 |
| 17q11.2del_NF1 | 10 |
| 17q12del | 9 |
| 17q12dup | 104 |
| 22q11.2del | 10 |
| 22q11.2dup | 294 |
| 2q11.2distal_del | 14 |

Supplementary Table 9. Number of individuals with each of the 33 P-CNVs

|  | MDD PRS | | Anxiety PRS | |
| --- | --- | --- | --- | --- |
|  | **OR** | **p-value** | **OR** | **p-value** |
| Help-seeking | 0.991 | 0.746 | 1.013 | 0.605 |
| Minimal | 0.970 | 0.498 | 0.972 | 0.521 |
| Primary care | 0.992 | 0.836 | 0.961 | 0.317 |
| Hospital | 1.001 | 0.891 | 0.984 | 0.698 |
| Medications | 1.103 | 0.031 | 1.074 | 0.100 |
| Initial self-report | 1.028 | 0.552 | 1.045 | 0.336 |
| MHQ self-report | 1.060 | 0.275 | 0.979 | 0.668 |
| CIDI-SF | 1.072 | 0.220 | 0.948 | 0.325 |

Supplementary Table 10. Association metrics of the interaction term of presence of NDD CNV with MDD and anxiety PRS for the eight definitions of internalising disorders.

**SUPPLEMENTARY FIGURES**


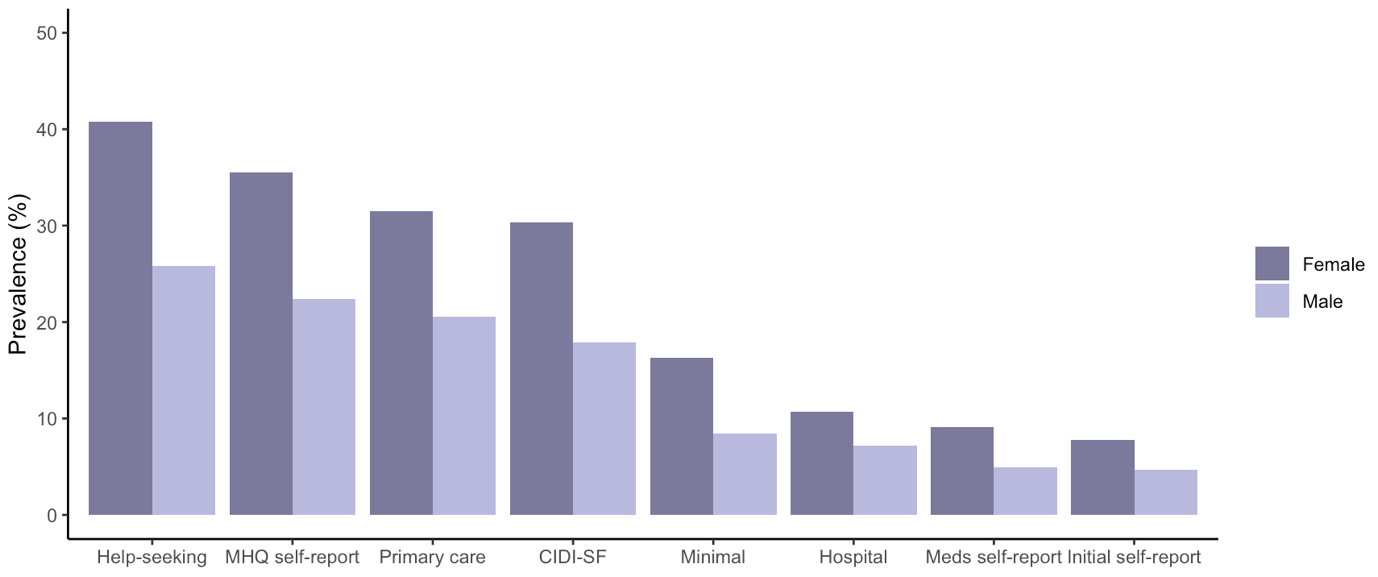


Supplementary Figure 1. Prevalence of each definition of internalising disorder by gender. For each definition, individuals with missing values were removed from the prevalence calculation.


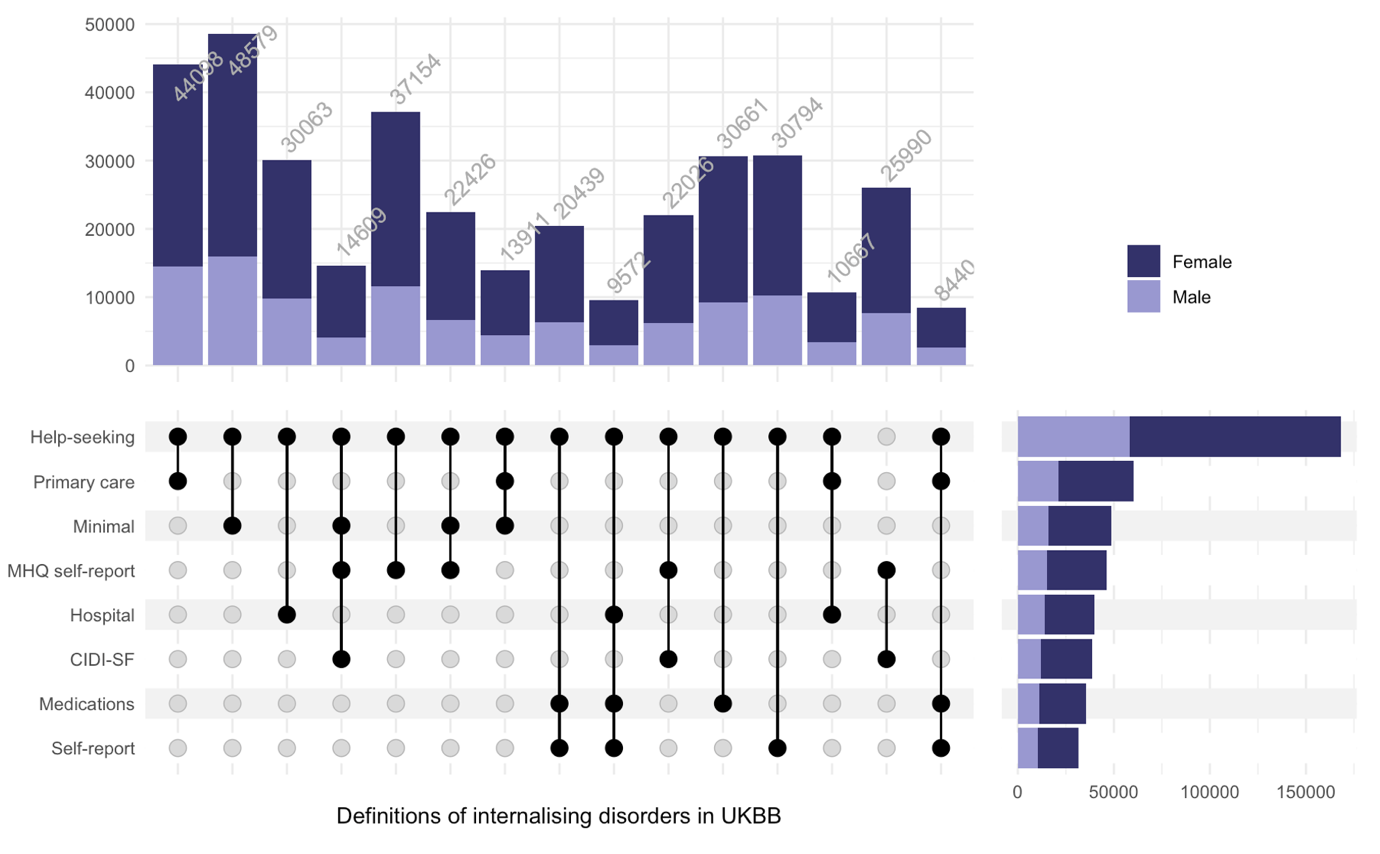


Supplementary Figure 2. Upset plot of the combinations of internalising disorder definitions.

*
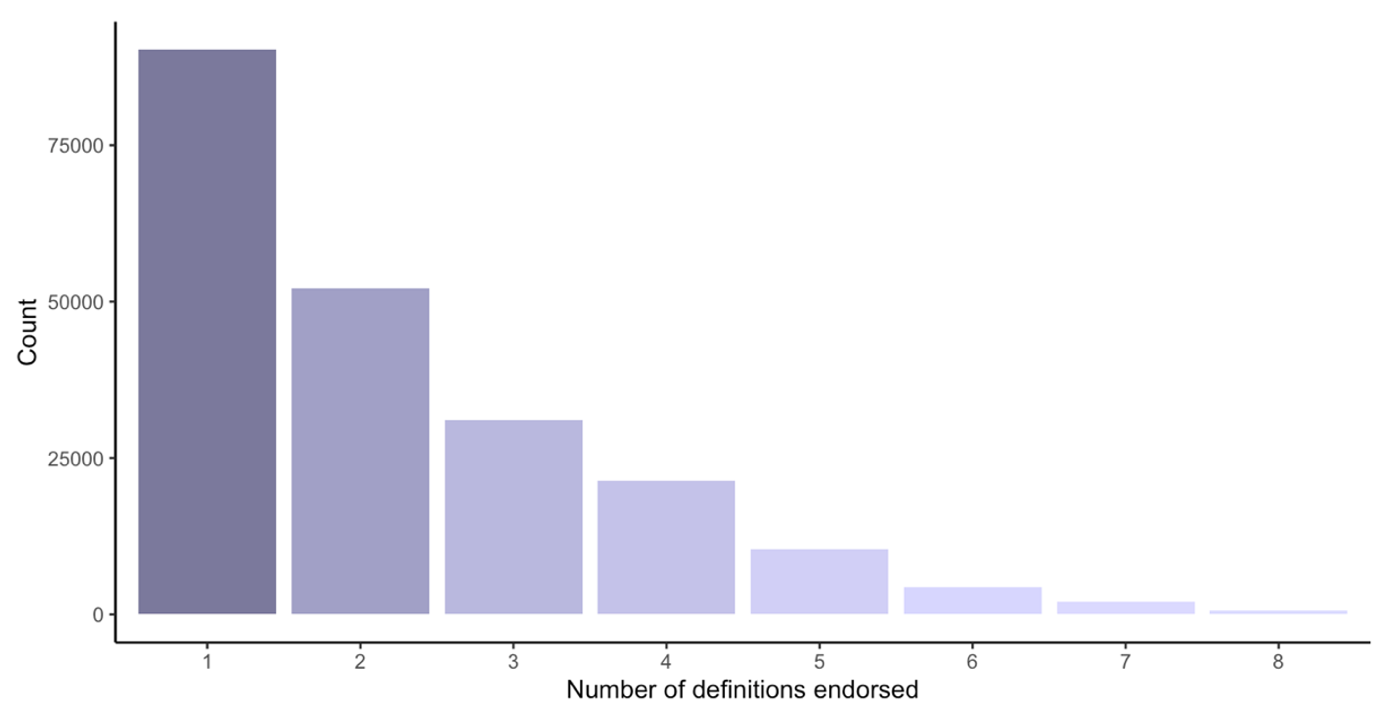
*

Supplementary Figure 3. Number of internalising disorder definitions endorsed by UKBB participants.


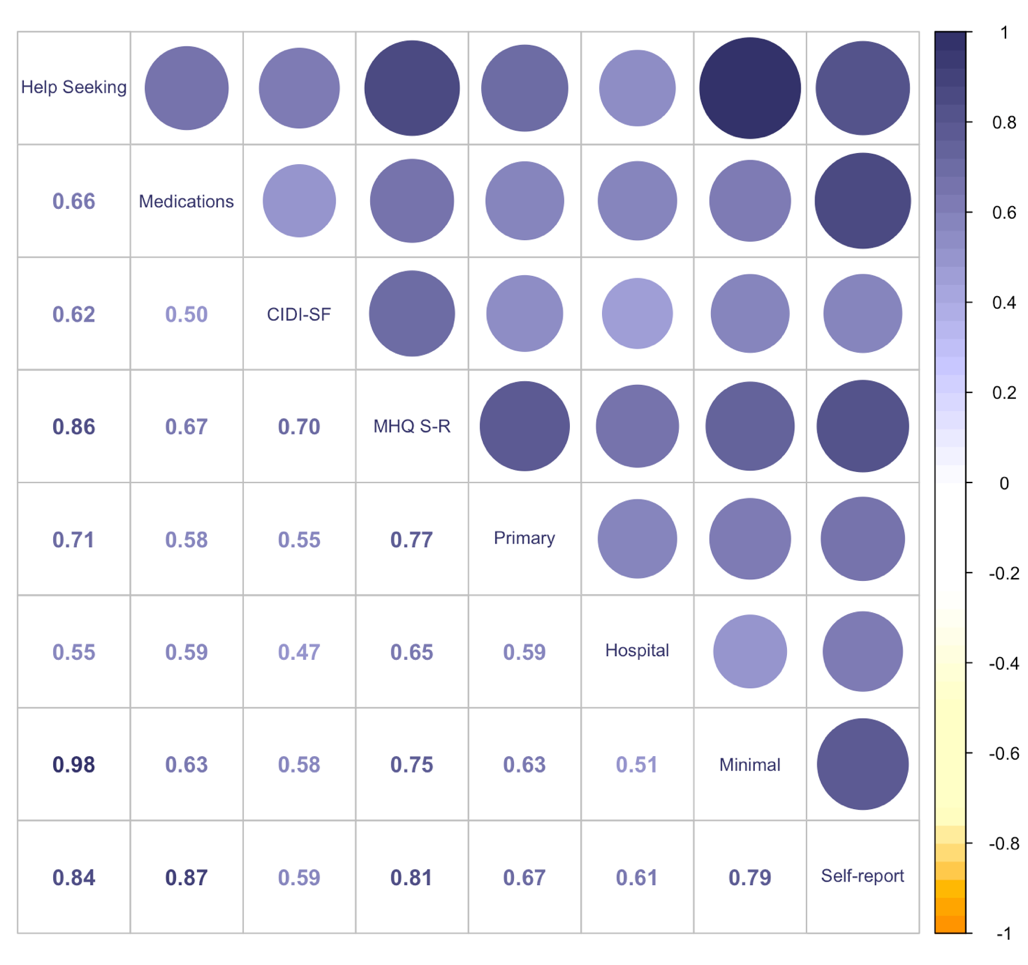


Supplementary Figure 4. Correlation plot of the eight internalising disorder definitions.


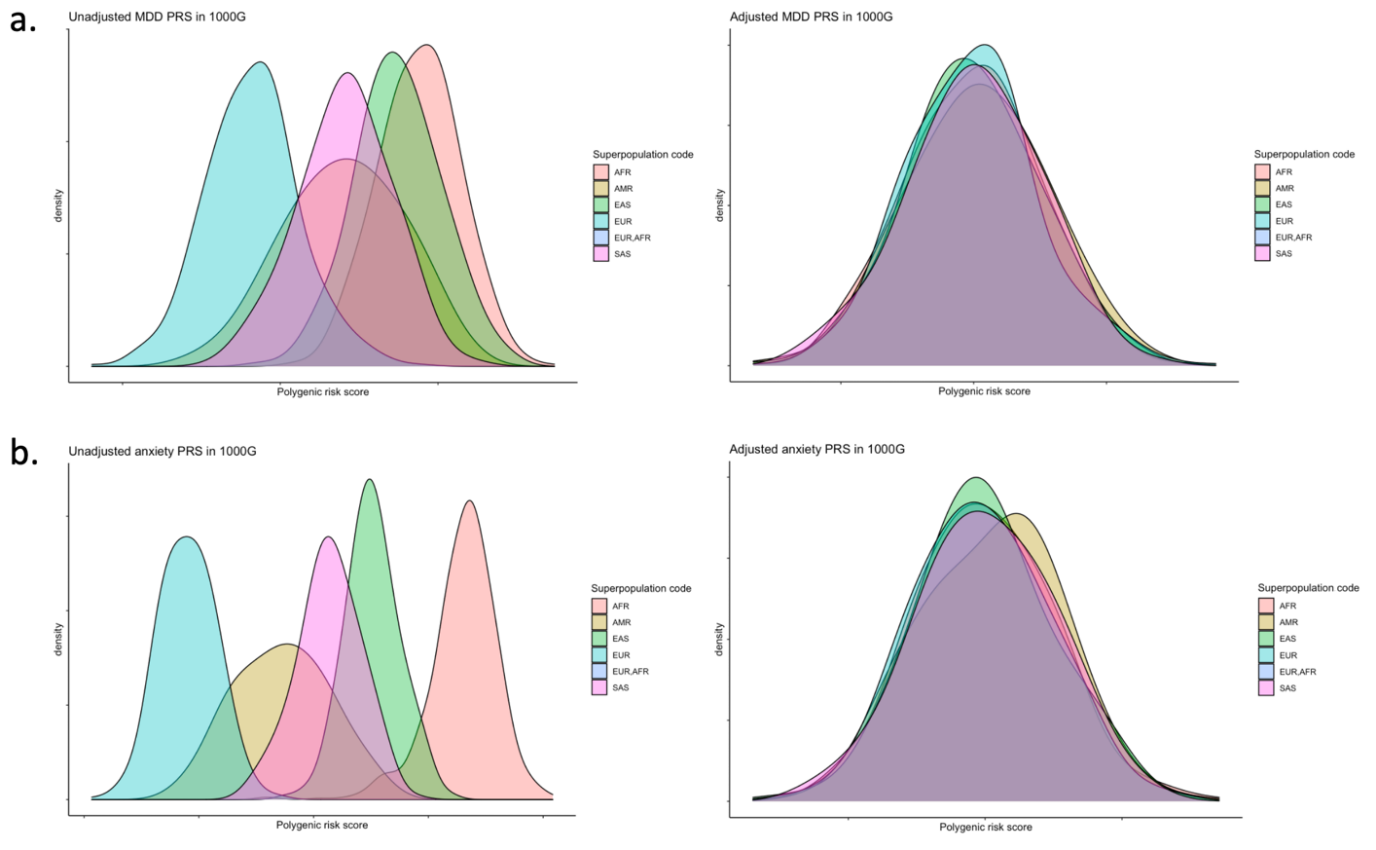


Supplementary Figure 5. Distribution of adjusted and unadjusted PRS of a. MDD and b. anxiety disorder in 1000 genomes populations.


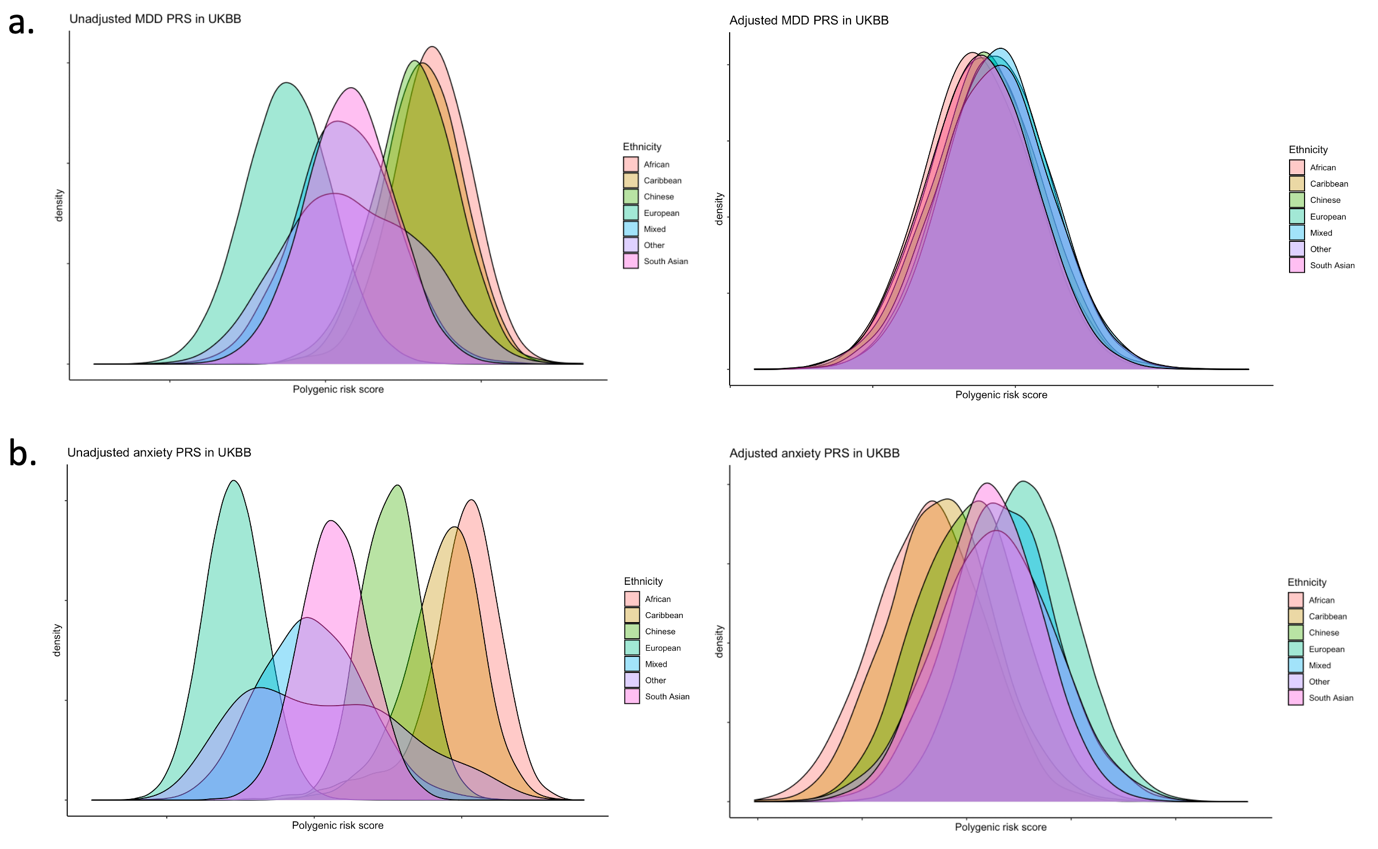


Supplementary Figure 6. Distribution of adjusted and unadjusted PRS of a. MDD and b. anxiety disorder in self-reported UKBB ethnicities.


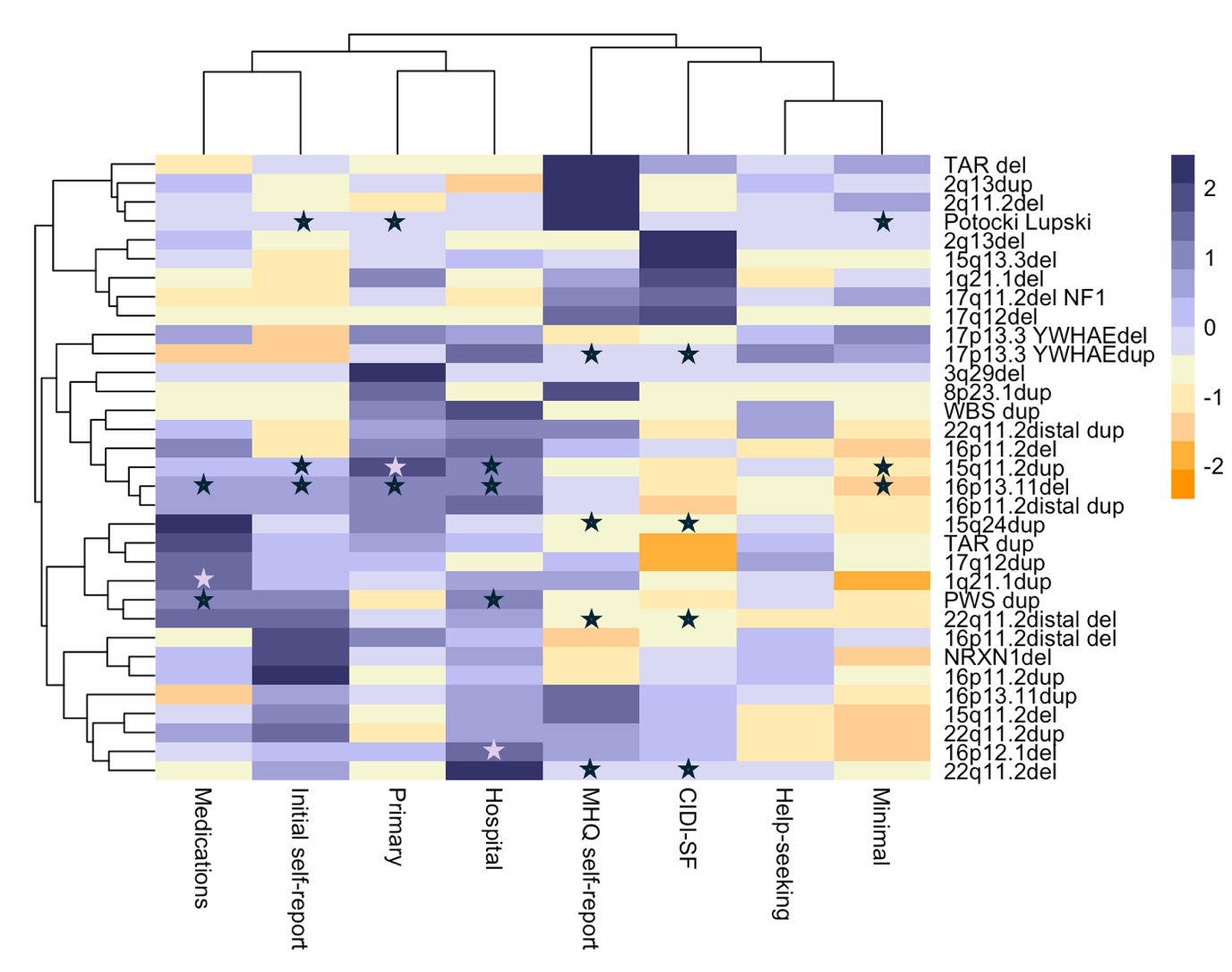


Supplementary Figure 7. Clustered heatmap of the scaled odds ratios of the logistic regression of the 33 NDD CNVs with the eight internalising disorder definitions. The stars indicate a significant association after Bonferroni correction for multiple testing (Bonferroni-corrected p-value threshold = 1.89x10^-4,^ N tests = 284).
